# Prevalence of Lower Back Pain (LBP) and Its Associated Risk Factors Among Alfaisal University Medical Students in Riyadh, Saudi Arabia: A Cross-Sectional Study

**DOI:** 10.3390/healthcare13131490

**Published:** 2025-06-22

**Authors:** Mohamad Behairy, Samir Odeh, Jouri Alsourani, Mohamad Talic, Sara Alnachef, Sadia Qazi, Muhammad Atif Mazhar, Hani Tamim

**Affiliations:** 1 College of Medicine, Alfaisal University, Riyadh 11533, Saudi Arabia; 2 Department of Anatomy, Alfaisal University, Riyadh 11533, Saudi Arabia

**Keywords:** low back pain, medical students, Saudi Arabia, epidemiology, ergonomics, musculoskeletal system

## Abstract

**Background**: Lower back pain (LBP) is defined as any recurring lumbar pain between the rib cage and the buttocks present at the time of the study. This study investigated the point prevalence, associated risk factors, and degree of disability of LBP among medical students at Alfaisal University, Riyadh, Saudi Arabia. **Methods**: A cross-sectional study evaluated 331 medical students using the Oswestry Disability Index (ODI; used to gauge LBP degree of disability) supplemented with demographic and lifestyle questions. The respondents were mostly first-year, female, and between the ages of 17 and 21 years. **Results**: Analysis uncovered that Female students, extended durations of phone usage, and those who did not exercise were more likely to experience LBP (*p* < 0.001; *p* = 0.042; *p* = 0.001). A higher degree of disability was associated with participants older than 21 years, who used their devices for extended periods, and who slept less (β = 0.170, *p* = 0.006). While most students experienced LBP (73.4%), the ODI revealed that the majority were not deemed disabled (56.9%). Factors associated with LBP prevalence were not necessarily associated with a higher degree of disability per the ODI. **Conclusions**: LBP is highly prevalent among medical students, with several associated risk factors. Female medical students remain a significant at-risk group. These findings highlight the need for a broader intervention against LBP, such as ergonomic and lifestyle improvements that consider a multitude of factors.

## 1. Introduction

Lower back pain (LBP) is the leading cause of disability worldwide and is responsible for more years lived with disability than any other condition [1]. In 2020, it affected an estimated 619 million people, and this number is projected to increase to 843 million by 2050, highlighting its growing impact on public health [2]. LBP is currently the leading cause of years lived with disability, affecting 619 million people and leading to almost a combined 69 million years of disability [2]. Globally, the mean prevalence of lower back pain ranges from 8% to 31%, according to various studies. LBP varies significantly across age, sex, and region [3]. It affects nearly 47% of the global population, and among those, 39% to 45% experience chronic symptoms that often require medical attention [4]. While it can impact individuals across all age groups and socioeconomic backgrounds, students, especially those in academically rigorous fields such as medicine, are disproportionately affected [2,5,6].

Lower back pain (LBP) is typically categorized as acute, subacute, or chronic based on the duration of symptoms [7,8]. Acute LBP lasts less than 1 month, subacute LBP lasts between 1 and 3 months, and chronic LBP persists for more than 3 months. According to the International Classification of Functioning, Disability, and Health (ICF), these categories can also be refined based on specific functional impairments, such as mobility deficits, coordination issues, or lower extremity-referred pain [9]. However, there is a growing debate about revising this time-based classification, as LBP is often recurrent rather than a single, isolated episode [9,10,11]. For instance, one emergency department study found that up to 44% of patients with acute LBP experienced recurrence [12]. Due to this complexity, the current study focused on the point prevalence of LBP at the time of data collection without further subclassification.

Upon deeper inspection, the LBP prevalence shows remarkable trends when examined regionally. In Saudi Arabia, the general population rates range from 63.8% to 89% [13]. Among Saudi adolescents aged 13–18 years, about 19% experience LBP, with 57.8% reporting symptoms in the past year [14]. In university students, studies have shown a prevalence of between 60% and 80% [2,15]. Sex differences persist, with females showing a 1–10% higher prevalence than males across various settings [16,17]. More specifically, medical students in Saudi Arabia report alarmingly high prevalence rates ranging from 80% to 94%, exceeding national and global averages and suggesting unique risks related to medical education [18,19,20].

Numerous risk factors contribute to the development of low back pain (LBP), especially those linked to physical, biomechanical, and lifestyle behaviors. Poor activation and endurance of trunk muscles can impair spinal stability, thereby increasing susceptibility to LBP [21]. Common contributors include weak core musculature, postural imbalances, movement coordination deficits, and prolonged sedentary behavior, especially prolonged sitting associated with study-based lifestyles, which disrupts spinal curvature and causes muscle strain [6,22]. Sitting for more than six hours without breaks significantly raises the risk of LBP by over fourfold [15]. Psychosocial factors, such as chronic stress, poor sleep, and low mood, exacerbate LBP severity [3,23]. Medical students are particularly vulnerable to musculoskeletal disorders due to prolonged sedentary behavior, poor posture, long hours of study, and significant psychological stress [24,25]. These factors not only contribute to the onset and persistence of LBP but also exacerbate its physical and psychological toll.

Research has linked chronic musculoskeletal pain in students to higher rates of depression and anxiety and lower quality of life [20,26,27]. In some cases, the burden of pain may lead to maladaptive coping behaviors, such as overuse of analgesics or even opioid misuse, increasing the risk of dependence and worsening mental health outcomes [28]. Thus, medical students face a unique dual burden: managing their susceptibility to LBP while being expected to prevent, recognize, and treat the condition in their future patients. This duality makes LBP both a personal health issue and a professional concern. Additionally, lifestyle and behavioral factors contribute to the burden on medical students. Their demanding curricula involve long hours of sedentary study, disrupted sleep patterns, and increased psychological stress, all of which are known to elevate LBP risk and severity [6]. The vast majority of LBP cases remain idiopathic or multifactorial, with serious underlying pathologies accounting for less than 1% of cases [29]. Psychosocial and occupational stressors are strongly correlated with disabling LBP, emphasizing the need for multidimensional management approaches [23].

Despite the expanding literature on LBP prevalence, significant gaps remain. Many studies have focused on prevalence without a detailed assessment of severity or disability using validated measures such as the Oswestry Disability Index, which looks at the degree of disability [15,17]. Furthermore, few studies have specifically differentiated medical students from other student populations or thoroughly investigated how sex, lifestyle, and academic behaviors influence LBP severity [19,30]. This is particularly important because medical students represent future healthcare providers, and their high LBP burden could impact productivity and healthcare delivery [31]. A better understanding of these factors is critical for informing prevention and intervention strategies tailored to this vulnerable group.

Given these existing gaps, there remains a significant need to better understand the role of various lifestyle and demographic factors—and their complex interplay—in contributing to LBP and disability, including potential concerns such as higher rates of disability among female students than among male students. This study aimed to address these knowledge gaps by examining both novel and previously studied factors implicated in lower back pain among medical students at Alfaisal University. Specifically, it will investigate the prevalence and severity of LBP and explore how factors like prolonged sitting, inadequate trunk muscle endurance, stress, and sex differences relate to the disease burden of this population.

To achieve this, the study will incorporate, among other items, the Oswestry Disability Index (ODI), a well-validated and widely used instrument among physicians and researchers for assessing the degree of disability caused by LBP [26,32,33,34]. This approach enables the exploration of statistically significant associations between demographic and lifestyle variables and LBP severity, providing a comprehensive understanding of the condition.

## 2. Methodology

### 2.1. Participants

This observational, cross-sectional study was conducted at Alfaisal University, Riyadh, Saudi Arabia, from March to April 2024. It involved medical students from Years 1–5 who consented to fill it out. The authors opted for a cross-sectional design due to its efficiency in collecting data from a large sample simultaneously, which is particularly advantageous given the busy schedules of medical students. This study adhered to the STROBE cross-sectional study guidelines.

Participants were eligible for this study if they met the following criteria:

1. Currently Enrolled Medical Students at Alfaisal University (Years 1–5, MBBS program)
2. Consented to the study IRB disclosure agreement

### 2.2. Sampling

The target population for this study comprised all medical students at Alfaisal University in Riyadh (approximately 1200 students). Multiple channels were used to maximize recruitment, including emails, broadcast messages, and in-person invitations through QR codes of the study form. Reminders were sent to encourage student participation.

To ensure statistical validity, the authors calculated the required sample size for a precision of 5% and a prevalence of 50% in a population of 1200 students. A minimum sample of 292 participants was obtained based on a 95% confidence interval (CI). This calculation was performed utilizing the sample size calculator available at https://www.calculator.net/sample-size-calculator.html (accessed on 1 December 2024). The authors collected responses using convenience/purposive sampling of the identified population (Alfaisal University Medical Students, Years 1–5).

### 2.3. Survey Instrument Outcomes

As the research team opted for an online method of data capture, the survey instrument comprised the following components:

1. Author-developed questions

These were specifically designed to gather demographic information and explore factors potentially associated with lower back pain (LBP) among medical students. They include information such as the study year, age, and sex.

2. A yes/no question on whether they were currently experiencing back pain, indicating the point prevalence of LBP.
3. A numerical scale was used to identify pain severity, scaled 1–10, where 0 was the least amount of pain, and 10 was the most severe pain experienced.
4. Oswestry Disability Index (ODI): The authors incorporated the chiropractic version of the ODI [32], a widely recognized tool for assessing LBP-related disability. This version consists of 10 questions, each with six possible answers scored from 0 to 5 [33]. The ODI score was calculated by summing the scores from all 10 questions, resulting in a total score ranging from 0 to 50.
5. This total score was used to determine the degree of LBP-related disability experienced by the respondents.
6. The ODI scores were interpreted as follows:
  0–4: No disability
  5–14: Mild disability
  5–24: Moderate disability
  25–34: Severe disability
  35–50: Complete disability
7. Additional validated questions: The authors also included questions on posture, medical advice-seeking behavior, device usage, and lifestyle choices drawn from previous studies [27,30,35].

The survey was initially piloted on a group of five students who filled it out with junk data and provided feedback on the wording and timing. Their input was essential for streamlining the UI of the survey to maximize the potential yield.

As the survey was to be completed by individuals using their devices, it was not possible to further examine respondents using physical tests or clinical confirmation of LBP diagnosis. However, by utilizing the previously-discussed components, the authors aimed to comprehensively assess the prevalence, severity, and risk factors of LBP among Alfaisal University medical students.

### 2.4. Data Collection

Data collection was conducted via Google Forms and adhered to the CHERRIES guidelines for online surveys (checklist attached, see Supplementary Materials). The form was a 30-question (20 standard questions, 10 ODI questions), 4-page, open-access electronic form. The form was distributed to students through multiple modalities, such as in-person pop-ups with QR codes, university-wide broadcast emails, and social media platforms, such as WhatsApp. The data collection period will last approximately one month, from March to April 2024. Participants were assured of the confidentiality of their responses, which were strictly for research purposes and guided by strict protocols from the university’s ethics board. The survey automatically excluded non-medical students, ensuring that only the target population, the students of the College of Medicine at Alfaisal University, could proceed to complete the survey. The survey allowed respondents to check their responses at the end.

Flowchart of total responses/data points collected. Over the course of the data-gathering period (March–April 2024), 337 students were initially queried via outreach methods. The inclusion criteria were current non-preparatory year medical students. Of these 337 students who provided responses, three responses were non-consensual and were discarded. Of the now-334 student responses, 3 were incomplete and discarded. Finally, 331 data points were analyzed in this study.

### 2.5. Hypothesis

To guide this investigation, the authors formulated the following hypothesis:

If *p* < 0.05, there was a statistically significant association between Lower Back Pain prevalence/degree of disability and examined risk factors in the medical students at Alfaisal University.

### 2.6. Statistical Analysis

Data was first stored, cleaned, and then analyzed using the IBM SPSS Statistics (version 29.0.2.0) software. Responses that did not consent to the disclosure agreement, in addition to incomplete or missing responses, were discarded. To conduct hypothesis testing, chi-squared analysis was utilized to identify associations between categorical demographics and the occurrence of lower back pain (LBP) and further analysis regarding the role of sex among significant determinants of LBP. To assess the relationship between mean Oswestry Disability Index (ODI) scores and demographic factors, we conducted an analysis of variance (ANOVA) and *t*-tests. Regression analysis was performed on variables like age, with adjustments made for variables. In this study, the authors set an alpha value of 0.05, considering results with *p* < 0.05 as significant.

### 2.7. Ethical Considerations

This study was conducted under the oversight of the Alfaisal Institutional Research Board (Protocol/Approval Code #20278, date of approval: 25 February 2024). The IRB periodically reviewed the data and materials relating to the course of the study.

The respondents were surveyed through an anonymous Google Forms survey tool that did not collect any form of identifier apart from the variables examined in the study. All data were stored on a secure server that was only accessible to the study team.

## 3. Results

### 3.1. Demographic Information of Participants and LBP Data

Over the course of the data collection period, 338 responses were gathered, with 336 respondents consenting to participate, of which only 331 provided complete responses. Thus, our final study sample comprised 331 students.

The respondents were from all five years of study at Alfaisal University and ranged in age from 17 to above 22. The average age of the respondents was 20 years, the average BMI was around 23, and the responses displayed a high female: male ratio. Most students surveyed were in the 17–21 age range (*n* = 278), with respondents primarily concentrated in the first two years of Alfaisal University medical school (44.7% and 25.4% for study years 1 and 2, respectively). A significant majority of respondents were in the normal BMI range (57.7%), followed by those in the overweight category (23.9%) and a small number of underweight and obese respondents (10.3% and 8.2%, respectively) (Table 1).

9A supermajority of surveyed students also spent long hours on their computer/laptop for various purposes (94%). Regarding phone usage, most students used their phones for an average of 2–5 h (42.9%), with a sizable minority using it for 5–8 h a day (33.8%). Additionally, the majority of students exercised (50.1%), with the largest percentage exercising for more than five hours. Most students were non-smokers, did not lift weights, and commuted to university in less than 30 min. Most respondents slept 4–6 h (55.0%), yielding an average sleep duration of 4.45 h. The sitting position of respondents was also questioned, which revealed that most students leaned forward when sitting in any situation (39.0). Regarding backpack usage and habits, the majority of respondents regularly wore a backpack (40.2%); however, the second-largest contingent of students following backpack wearers was the group of students who wore a shoulder bag or a crossbody (34.7%) (Table 1).

Regarding lower back pain data, a significant majority of students admitted to suffering from lower back pain (LBP) in any capacity (73.4%, *n* = 253). On a scale of 1–10, where 10 is the most severe, the average LBP severity was 4.61 ± 2.16 (Table 2).

### 3.2. Exploration of LBP Presence and Disability Risk Factors

Table 3 presents the results of the chi-square analysis. Various lifestyle and demographic factors were analyzed with regard to the presence or absence of lower back pain in participants.

Regarding the demographic profile of the participants, the analysis revealed a statistically significant relationship between sex and the presence of LBP. LBP presence was strongly skewed towards female rather than male participants (*p* ≤ 0.001). No other statistically significant relationships were found in the demographic profiles of the participants.

Numerous statistically significant relationships exist surrounding the lifestyle factors and behaviors of the participants. For instance, exercise and LBP also displayed a statistically significant relationship; however, the duration of exercise did not seem to be associated (*p* = 0.001 and 0.287, respectively). Regarding exercise, lifting weights also displayed strong significance with back pain, with a larger number of weight-lifting participants possessing back pain than their counterparts who did not (*p* ≤ 0.001). The duration of average daily phone use was also an important lifestyle factor noted in the analysis, with a slight relationship (*p* = 0.042). Regarding the sitting position of participants, there was a strong relationship between it and LBP, with most participants leaning either forward or backward (*p* = 0.014). Additionally, the participants’ habit of wearing backpacks or similar items displayed a strong relationship with LBP (*p* = 0.001). Most participants either wore a backpack or a shoulder bag/crossbody to university.

**Figure 1:**
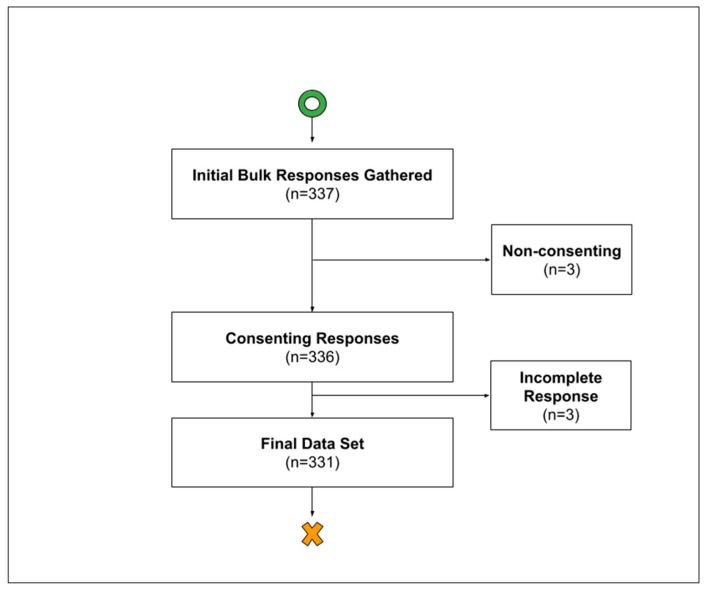
Responses gathered at each stage of the study.

Following the chi-square analysis of how each factor related to the presence or absence of lower back pain, a subsequent analysis focused on sex differences among the statistically significant variables uncovered. (*p* < 0.05). Sex was cross-tabulated with factors such as exercise, weight lifting, average phone usage duration, sitting position, and backpack use. Figure 1 illustrates these sex-specific trends.

**Figure 2:**
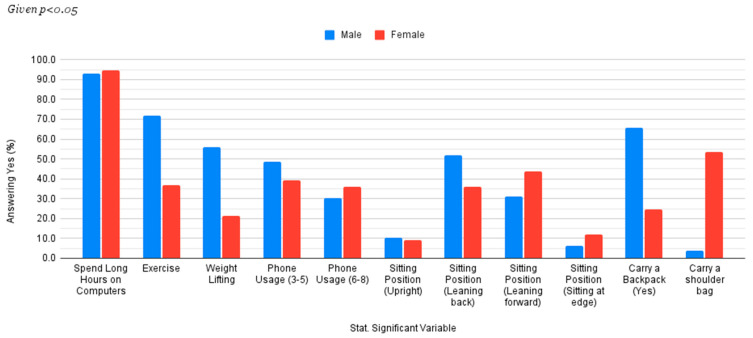
Sex differences in statistically significant lifestyle factors and behaviors (differences >5% excluded). Note: Figure of the sex differences among statistically significant lifestyle and behavioral factors uncovered following the chi-square analysis. The figure represents data taken from Table 4 but visualized to further understand the role that sex plays in these factors. This analysis was undertaken following previous literature pointing to potential relationships. The results show that the largest sex differences exist in exercising, seating posture (leaning back, leaning forward), wearing a backpack, and wearing a shoulder bag/crossbody.

The results revealed distinct patterns between male and female students (Table 4, Figure 2. Both sexes reported high rates of long computer usage, with 92.8% of males and 94.7% of females spending extended periods on computers. However, significant disparities were observed in physical activity. Male students showed higher engagement in exercise (72.0% exercising regularly) than female students (only 36.9%). Similarly, weightlifting was more prevalent among males (56.0%) than females (21.4%).

Regarding daily phone usage, a higher percentage of male students (48.8%) reported using their phones for 3–5 h compared with female students (39.3%). However, for 6–8 h of daily usage, the trend reversed slightly, with 35.9% of females versus 30.4% of males.

Sitting posture analysis indicated sex-specific preference. Male students were more likely to lean back (52.0%) than female students (34.5%), while a higher proportion of female students (43.7 %) tended to lean forward when seated compared to male students (31.2%).

A stark contrast was observed in terms of bag preferences. Most male students (65.6%) opted for backpacks, while only 24.8% of female students did so. Conversely, shoulder bags or crossbody bags were overwhelmingly preferred by female students (53.4%) compared with male students (4.0%).

The Oswestry Disability Index (ODI) was used to assess the level of disability that students admitted to suffering from Lower Back Pain (LBP). As depicted in Table 5, the majority of respondents (56.9%, *n* = 144) were classified as having “No Disability”. A substantial proportion (37.2%, *n* = 94) fell into the “Mild Disability” category. A smaller fraction of participants were categorized with more significant impairment: 4.3% (*n* = 11) showed “Moderate” disability, while only 1.6% (*n* = 4) were classified as having “Severe Disability.” These findings suggest that while LBP was prevalent in the study population, the impact on daily functioning, as indicated by the severity levels uncovered by the ODI, was relatively minimal for most participants. Over 94% of the respondents experienced either no disability or only mild disability, indicating that the majority of individuals with LBP in this study were able to manage their daily activities with little or no significant impairment (Table 5).

The respondents’ Oswestry Disability Index (ODI) scores were also analyzed with regard to the various demographic and lifestyle factors using Analysis of Variance (ANOVA) and independent *t*-test statistical analyses, yielding mean ODI scores for each demographic and lifestyle factor in addition to the degree of statistical significance of those relationships. Most categories of factors were correlated with a mean ODI between 5 and 14 points, categorizing them as “low-disability, ” with small numbers of specific factors with mean scores below 5 leading to a categorization of “no-disability.”

The age range of the respondents displayed statistically significant variance (*p* = 0.08) between participants aged 17 to 21 years and those aged 22 years and over, with the former having an average ODI score of 4.86 (thus categorizing them as “No Disability” per the Oswestry Disability Index) and the latter 7.14% (“Mild Disability”). No other demographic factors showed statistically significant differences in ODI scores.

Regarding demographic factors, several displayed valid relationships with average ODI scores. First, participants who admitted to spending long hours on their devices displayed markedly higher Oswestry index scores than those who did not (5.47 vs. 1.57; *p* = 0.008). Additionally, daily phone usage duration was shown to display statistically significant variance among participants; those who utilized their phones for less than an hour or more than 8 h (10.75 for those >1 h and 7.21 for those 8+; *p* = 0.028). Those who used their phones for intermediate durations had similar scores. The average sleep time of the participants was also shown to be statistically significant with regard to the Mean ODI score, possessing an inverse relationship with ODI scores (*p* = 0.023). The results are summarized in Table 6.

To further explore the relationship between age (as a continuous variable) and the Oswestry Disability Index (ODI) score of the participants, a linear regression analysis was conducted with adjustment for the other statistically significant variables impacting the Oswestry Disability Index Score (phone usage duration, spending long hours on devices, and sleep duration). Following adjustment of other statistically significant determinants of ODI scores uncovered through ANOVA and t-test analysis, the model displayed that age did indeed possess a statistically significant relationship on ODI score (*p* = 0.026). Age was positively correlated with the Oswestry Disability Index score (*β* = 0.137), indicating that as age increases, ODI scores also increase. Additionally, the regression model revealed that sleep duration had a negative relationship with the ODI score of participants (*β* = −0.140; *p* = 0.025), suggesting that sleeping more decreases the ODI score, while spending long hours increases the ODI score due to the positive relationship (*β* = 0.170; *p* = 0.006). The duration of phone use did not meet the alpha criterion of the study (*p* > 0.05). The results are summarized in Table 7.

## 4. Discussion

This study highlights that a considerable number of medical students experience lower back pain (LBP), with the majority reporting mild to moderate pain. These findings among medical students in this study align with previous research from different geographical locations, including studies from Bangladesh and Saudi Arabia [17,30,36,37], which similarly identified a substantial proportion of medical students experiencing LBP. However, some previous investigations have reported notably lower prevalence rates, such as those conducted at Taif University in Saudi Arabia and Serbia [18,38]. Such discrepancies could potentially stem from variations in lifestyle habits, cultural differences, or methodological differences, such as the study design.

Female sex, engagement in physical activity, weightlifting habits, and carrying backpacks were significantly associated with LBP. Notably, factors such as BMI, year of study, smoking habits, and sitting posture were not significantly associated with LBP prevalence. It is noteworthy that the majority (70.8%) of students with LBP did not participate in weightlifting, even though some engaged in exercise. This could hint at the potential protective nature of weightlifting against LBP, with one possible explanation being that certain weightlifting techniques could help strengthen the back and core muscles, potentially reducing or preventing LBP [23].

This study also indicated a strong and significant association between LBP and sex, showing that more female than male medical students were affected. This is consistent with a previous study that also found a strong association between sex and LBP, with female students being more prone to LBP than male students [38]. Female risk factors for LBP have not been thoroughly examined in the past, with previous studies stating that females could face higher risks of LBP due to the nature of female-specific considerations (hormonal fluctuations) [30,38,39,40].

To further understand why females are at a higher risk of having LBP, the authors of this study performed a cross-tabulation between sex and all statistically significant variables. They found that 78.8% of participants who did not exercise were females and that 74.7% of participants who did not lift weights were also females. Regarding sitting postures, there was no significant difference between males and females, as 90% of participants of each sex reported that they sat in postures found not to be ideal. Similar trends have been observed in studies such as that by Vujcic et al., who found a sex association with posture, among other factors [18]. Ultimately, insufficient exercise and weightlifting, in addition to potential extraneous factors such as hormonal impact, could be the main reasons why LBP is generally more prevalent in female students.

Interestingly, this study identified an inconsistency between pain perception and disability, as indicated by the ODI scores. A considerable proportion of students who reported moderate pain simultaneously reported minimal or no disability, an observation aligned with previous studies [17,18,22,30]. Factors such as age, sleep duration, and prolonged use of electronic devices were strongly correlated with higher disability scores. This disconnect between LBP risk and disability severity was seen with the risk factor of prolonged device use, which, while not associated with overall LBP prevalence in our study, was strongly related to higher disability. In addition, the regression analysis confirmed a similar relationship, with device usage exhibiting a positive correlation with the ODI score. This finding is particularly relevant given the increasing reliance on electronic devices in medical education, emphasizing the need to address ergonomic considerations in virtual learning environments [5,41].

These results underscore the importance of targeted interventions, particularly emphasizing physical activity and ergonomics and addressing female-specific risk factors to effectively mitigate LBP prevalence and its associated disabilities among medical students. Further research is encouraged to delve deeper into these associations and evaluate the effectiveness of preventive strategies.

### Limitations, Strengths, and Future Directions of the Study

Regarding the limitations of this study, there are several avenues that can be improved in future investigations. First, the study had a cross-sectional design and, therefore, could be liable to both causality and selection bias. Causality bias can limit the study in that it is not possible to conclude that participants experience LBP more or less due to medical school. On the other hand, selection bias might have affected this study in that participants who suffered from LBP might have been more open to participating in this study while those who did not suffer from LBP inclined to abstain from participating in the study. Secondly, due to the nature of the study, it was not possible to validate an LBP diagnosis with a clinician or radiological methods, leading the authors to rely on respondent self-diagnosis. Additionally, the study did not consider the characteristics of pain, such as duration and nature.

The strengths of this study primarily arise from the fact that it examined a large sample size from Alfaisal University and used a validated tool (Oswestry Disability Index) to analyze the characteristics of Lower Back Pain. Additionally, including validated questions and examining a large number of demographic and lifestyle variables allowed the exploration of numerous relationships between them and LBP.

Regarding study generalizability, this study entailed strict inclusion criteria so that the population being studied was clearly defined. In addition, this study yielded results that are consistent with those of similar studies, suggesting internal validity. Conversely, the study might possess limited external validity when extrapolated to other cohorts in the healthcare field (residents, specialists, and nurses, among others) due to the selection criteria.

Further investigation into this topic should look to go deeper into all aspects of LBP (severity, temporal characteristics, and degree of disability) with regard to a multitude of factors. This may include studies employing approaches other than cross-sectional studies. Further investigation can also look at modalities of objective measurements, such as imaging studies or physical examination, to rule out any issues arising from self-reporting a diagnosis. The novel findings uncovered in this study offer sources of expansion, such as the factors predisposing female medical students to increased disability rates (via hormonal studies) and the complex interplay between exercise participation/duration and the potential protective mechanism against LBP.

## 5. Conclusions

In conclusion, this study enabled the construction of a profile of those who suffer from LBP among medical students at Alfaisal University. Lower back pain was reported by 73.4% of the surveyed Alfaisal University Medical students, with an average severity of 4.61 ± 2.16. These students are mostly female, between the ages of 17 and 21, and use their devices for long hours, among other demographic trends.

Statistical analyses of the data supported the initial hypothesis that there are numerous relationships between various demographic/behavioral factors and LBP preponderance. The study found that LBP was correlated with female, sedentary, and computer-bound students. Interestingly, factors predisposing students to LBP do not necessarily predispose them to disability (and vice versa). Factors associated with a higher degree of disability included being above the age of 21 years, spending long hours on computers, using their phone for more than 8 h, and sleeping very little.

As LBP is a complex and often idiopathic condition, these results hold promise for narrowing down its potential determinants in critical cohorts. Based on the study’s findings, the authors propose the following recommendations to reduce the prevalence and severity of LBP among medical students:

Lessen the need for students to spend long hours on their devices that may result in less-than-ideal postures.

Encouraging proper posture and investing in better seating that takes into account ergonomics in university halls, laboratories, and general areas is essential.

Including scheduled breaks in the curriculum and increasing the focus on physical activity to combat a sedentary lifestyle, particularly for high-risk LBP cohorts such as female medical students, is recommended.

Strength training and core training should be promoted by providing opportunities for physical activity, whether by investing in fitness centers or increasing their accessibility to students.

Guidelines should be set to tackle long durations of device usage and bad seating postures among medical students and residents.

## Figures and Tables

**Table 1:** Demographics and lifestyle habits of survey respondents.

Variable		Frequency (%, 95% CI)
Age, mean ± SD (95% CI) (years)		20.01 ± 1.81 (19.82, 20.21)
Age (years)	17–21	278 (84.0 [79.7, 87.5])
	22+	53 (16.0 [12.5, 20.3])
Sex	Female	206 (62.2 [56.9, 67.3])
	Male	125 (37.8 [32.7, 43.1])
BMI, mean ± SD (95% CI) (kg/m^2^)		23.63 ± 4.60 (23.14, 24.13)
BMI Category	Underweight (<18.5)	34 (10.3 [7.4, 14.0])
	Normal (18.5–24.9)	191 (57.7 [52.3, 62.9])
	Overweight (25.0–29.9)	79 (23.9 [19.6, 28.7])
	Obese (30+)	27 (8.2 [5.7, 11.6])
Year In Medical School	Year 1	148 (44.7 [39.4, 50.1])
	Year 2	84 (25.4 [21.0, 30.3])
	Year 3	52 (15.7 [12.2, 20.0])
	Year 4	26 (7.9 [5.4, 11.3])
	Year 5	21 (6.3 [4.19, 9.5])
Reported Self-Defined Back Pain Before Medical School	No	161 (48.6 [43.3, 54.0])
	Yes	170 (51.4 [46.0, 56.7])
Spend Long Hours on Computers	No	20 (6.0 [3.9, 9.2])
	Yes	311 (94.0 [90.9, 96.1])
Exercise	No	165 (49.8 [44.5, 55.2])
	Yes	166 (50.2 [44.8, 55.5])
Weekly exercise (h) if Exercise	1–2	47 (28.3 [22.0, 35.6])
	3–5	53 (31.9 [25.3, 39.4])
	5+	66 (39.7 [32.6, 47.4])
Lift Any Weights During Exercise	No	217 (65.6 [60.3, 70.5])
	Yes	114 (34.4 [29.5, 39.7])
Smoke	No	296 (89.4 [85.6, 92.3])
	Yes	35 (10.6 [7.7, 14.4])
Average daily phone usage (h)	<1	4 (1.2 [.04, 3.1])
	1–2	31 (9.4 [6.68, 13.0]
	2–5	142 (42.9 [37.7, 48.3])
	5–8	112 (33.8 [29.0, 39.1])
	8+	42 (12.7 [9.5, 16.7])
University Commute Length (Minutes)	Less than 30	180 (54.4 [49.0, 59.7])
	More than 30	151 (45.6 [40.3, 51.0])
Average sleep time (h)	4>	24 (7.3 [4.9, 10.6])
	4–6	182 (55 [49.6, 60.3])
	6–8	103 (31.1 [26.4, 36.3])
	8+	22 (6.6 [4.4, 9.9])
Sitting Position	Upright	33 (10 [7.2, 13.7])
	Leaning Back	136 (41.1 [35.9, 46.5])
	Leaning forward	129 (39 [33.9, 44.3])
	Sitting at edge	33 (10 [7.19, 13.7])
Wear Backpack	Yes	133 (40.2 [35.0, 45.5])
	Sometimes	57 (17.2 [13.5, 21.7])
	No	26 (7.9 [5.42, 11.3])
	Carry shoulder bag or crossbody	115 (34.7 [29.8, 40.0])

**Table 2:** Point prevalence and severity of lower back pain in respondents.

Variable		Frequency (%)
Experience Back Pain	No Yes	78 (23.6) 253 (73.4)
Severity of Back Pain, mean ± SD (95% CI) (1–10)	4.61 ± 2.16 (4.34, 4.87)	

**Table 3:** Chi-square analysis of lower back pain and various factors.

Variable		Do Not Suffer Back Pain (%)	Suffer Back Pain (%)	*p*-Value	Effect Size (Phi/Cramer’s)
Age	17–21	50 (64.1)	75 (29.6)	0.218	0.068
	22+	28 (35.9)	178 (70.4)		
Sex	Male	69 (88.5)	209 (82.6)	<0.001 *	0.302
	Female	9 (11.5)	44 (17.4)		
BMI	Underweight	10 (12.8)	24 (9.5)	0.836	0.051
	Normal	45 (57.7)	146 (57.7)		
	Overweight	17 (21.8)	62 (24.5)		
	Obese	6 (7.7)	21(8.3)		
Year of Study	Year 1	35 (44.9)	113 (44.7)	0.302	0.121
	Year 2	21 (26.9)	63 (24.9)		
	Year 3	16 (20.5)	36 (14.2)		
	Year 4	4 (5.1)	22 (8.7)		
	Year 5	2 (2.6)	19 (7.5)		
Reported Self-Defined Back Pain Before Medical School	No	46 (59.0)	115 (45.5)	0.037 *	0.115
	Yes	32 (41.0)	138 (54.5)		
Spend Long Hours on Computers	No	6 (7.7)	14 (5.5)	0.484	0.038
	Yes	72 (92.3)	239 (94.5)		
Exercise	No	26 (33.3)	139 (54.9)	0.001 *	0.183
	Yes	52 (66.7)	114 (45.1)		
Weekly exercise (h)	1–2	11 (20.8)	37 (32.5)	0.287	0.122
	3–5	18 (34.0)	35 (30.7)		
	5+	24 (45.3)	42 (36.8)		
Lift Any Weights During Exercise	No	38 (48.7)	179 (70.8)	<0.001 *	0.197
	Yes	40 (51.3)	74 (29.2)		
Smoke	No	70 (89.7)	226 (89.3)	0.917	0.006
	Yes	8 (10.3)	27 (10.7)		
Average daily phone usage (h)	<1	0 (0.0)	4 (1.6)	0.042 *	0.173
	1–2	5 (6.4)	26 (10.3)		
	2–5	45 (57.7)	97 (38.3)		
	5–8	20 (25.6)	92 (36.4)		
	8+	8 (10.3)	34 (13.4)		
University Commute Length (Minutes)	Less than 30	48 (61.5)	132 (52.2)	0.147	0.080
	More than 30	30 (38.5)	121 (47.8)		
Average sleep time (h)	4>	2 (2.6)	22 (8.7)	0.272	0.109
	4–6	46 (59.0)	136 (53.8)		
	6–8	26 (33.3)	77 (30.4)		
	8+	4 (5.1)	18 (7.1)		
Sitting Position	Upright	7 (9.0)	26 (10.3)	0.014 *	0.180
	Leaning Back	44 (56.4)	92 (36.4)		
	Leaning forward	20 (25.6)	109 (43.1)		
	Sitting at edge	7 (9.0)	26 (10.3)		
Wear Backpack	Yes	8 (10.3)	18 (7.1)	0.001 *	0.225
	Sometimes	10 (12.8)	47 (18.6)		
	No	45 (57.7)	88 (34.8)		
	Carry shoulder bag or crossbody	15 (19.2)	100 (39.5)		

* *p*-score is deemed statistically significant.

**Table 4:** Further analysis of sex with relation to factors associated with lower back pain (*p* < 0.05).

Variable		Frequency of Males (%, 95% CI)	Frequency of Females (%, 95% CI)
Spend Long Hours on Computers	No	9 (7.2 [3.8, 13.1])	11 (5.3 [3.01, 9.3])
	Yes	116 (92.8 [86.9, 96.2])	195 (94.7 [90.7, 97.0])
Exercise	No	35 (28.0 [20.9, 36.4])	130 (63.1 [56.3, 69.4])
	Yes	90 (72.0 [63.6, 79.1])	76 (36.9 [30.6, 43.7])
Lift Any Weights During Exercise	No	55 (44.0 [35.6, 52.8])	162 (78.6 [72.5, 83.7])
	Yes	70 (56.0 [47.2, 64.4])	44 (21.4 [16.3, 27.5])
Average daily phone usage (h)	<1	2 (1.6 [0.4, 5.6])	2 (1.0 [0.2, 3.5])
	1–2	9 (7.2 [3.8, 13.1])	22 (10.7 [7.2, 15.6])
	3–5	61 (48.8 [40.2, 57.5])	81 (39.3 [32.9, 46.1])
	6–8	38 (30.4 [23.0, 38.9])	74 (35.9 [29.7, 42.7)
	8+	15 (12.0 [0.0741, 0.189])	27 (13.1 [0.0917, 0.184])
Sitting Position	Upright	13 (10.4 [6.2, 17.0])	20 (9.7 [6.4, 14.5])
	Leaning Back	65 (52.0 [43.3, 60.6])	71 (34.5 [28.3, 41.2])
	Leaning forward	39 (31.2 [23.7, 39.8])	90 (43.7 [37.1, 50.5])
	Sitting at Edge	8 (6.4 [3.3, 12.1])	25 (12.1 [8.4, 17.3])
Carry a Backpack	No	11 (8.8 [4.9, 15.1])	15 (7.3 [4.4, 11.7])
	Sometimes	27 (21.6 [15.3, 29.6])	30 (14.6 [10.4, 20.0])
	Yes	82 (65.6 [56.9, 73.4)	51 (24.8 [19.4, 31.1])
	Instead carry shoulder bag or crossbody	5 (4.0 [1.72, 9.0])	110 (53.4 [46.6, 60.1])

**Table 5:** Oswestry Disability Index Categorization of LBP: Identifying Respondents.

Oswestry Index Degree of Disability	Frequency (%, 95% CI)
No Disability	144 (56.9 [50.8, 62.9])
Mild Disability	94 (37.2 [31.4, 43.3])
Moderate	11 (4.3 [2.4, 7.6])
Severe Disability	4 (1.6 [0.6, 3.9])

**Table 6:** Comparison of mean LBP-identifying respondent ODI score by demographic and behavioral variables.

Variable		Mean ODI Score ± SD	*t/f*-Value **	*p*
Age	17–21	4.86 ± 4.87	−1.988	0.008 *
	22+	7.14 ± 7.29		
Sex	Male	4.68 ± 5.65	−1.099	0.778
	Female	5.50 ± 5.32		
BMI	Underweight	5.46 ± 5.84	1.838	0.141
	Normal	4.62 ± 4.76		
	Overweight	6.13 ± 6.30		
	Obese	6.86 ± 6.21		
Year of Study	Year 1	5.12 ± 5.02	0.639	0.635
	Year 2	5.56 ± 5.54		
	Year 3	4.81 ± 5.54		
	Year 4	4.50 ± 4.06		
	Year 5	6.84 ± 8.08		
Reported Self-Defined Back Pain Before Medical School	No	5.23 ± 5.29	−0.059	0.765
	Yes	5.28 ± 5.55		
Spend Long Hours on Computers	No	1.57 ± 1.51	−2.648	0.008 *
	Yes	5.47 ± 5.49		
Exercise	No	5.23 ± 5.06	−0.86	0.170
	Yes	5.29 ± 5.86		
Weekly exercise (h)	1–2	4.62 ± 3.80	2.995	0.054
	3–5	7.26 ± 7.30		
	5+	4.24 ± 5.73		
Lift Any Weights During Exercise	No	5.46 ± 5.59	0.918	0.573
	Yes	4.77 ± 5.00		
Smoke	No	5.04 ± 5.33	−1.890	0.763
	Yes	7.11 ± 5.93		
Average daily phone usage (h)	<1	10.75 ± 6.90	2.765	0.028 *
	1–2	5.15 ± 4.93		
	2–5	5.18 ± 5.05		
	5–8	4.41 ± 4.52		
	8+	7.21 ± 7.86		
University Commute Length (Minutes)	Less than 30	5.08 ± 5.77	−0.554	0.388
	More than 30	5.45 ± 5.03		
Average sleep time (h)	4>	8.23 ± 7.13	3.218	0.023 *
	4–6	5.37 ± 5.58		
	6–8	4.23 ± 4.35		
	8+	5.17 ± 4.96		
Sitting Position	Upright	4.58 ± 4.53	1.140	0.333
	Leaning Back	5.86 ± 6.54		
	Leaning forward	4.70 ± 4.37		
	Sitting at edge	6.15 ± 5.82		
Wear Backpack	Yes	6.61 ± 6.85	1.132	0.337
	Sometimes	5.72 ± 5.38		
	No	4.49 ± 4.81		
	Carry shoulder bag or crossbody	5.47 ± 5.65		

Note: * *p*-score deemed statistically significant; ** *t*-test score displayed for variables with only two outcomes, *f*-value for those with >2.

**Table 7:** Regression analysis of participant age on Oswestry Index Score, adjusted for spending long hours on devices, avg. duration of sleep and avg. duration of phone usage.

Variable **	Std. Beta (95% CI)	Std. Error	*p*
Constant	n/a (n/a)	4.128	0.207
Age (cont.)	0.137 (0.048, 0.763)	0.182	0.026 *
Duration of sleep (Ranges)	−0.140 (−1.91, −0.132)	0.451	0.025 *
Spend Long Hours on Computer (y/n)	0.170 (1.172, 6.895)	1.453	0.006 *

* *p*-score deemed statistically significant; ** The risk factor of Average daily phone usage duration results not included in the table as it did not meet alpha (*p* = 0.934).

## Data Availability

All data generated or analyzed during this study are included in this published article.

## Supplementary Materials

The following supporting information can be downloaded at: https://www.mdpi.com/article/10.3390/healthcare13131490/s1. File S1: CHERRIES Checklist. Reference [42] is cited in the Supplementary Materials.

## Abbreviations

LBP	Lower back pain
ODI	Oswestry Disability Index
